# Dichloridobis(5-*m*-tolyl-1,3,4-thia­diazol-2-ylamine-κ*N* ^3^)zinc(II)

**DOI:** 10.1107/S160053680801057X

**Published:** 2008-04-23

**Authors:** Bin Wang, Rong Wan, Li-He Yin, Feng Han, Jin-Tang Wang

**Affiliations:** a Department of Applied Chemistry, College of Science, Nanjing University of Technology, No. 5 Xinmofan Road, Nanjing 210009, People’s Republic of China

## Abstract

In the mol­ecule of the title compound, [ZnCl_2_(C_9_H_9_N_3_S)_2_], the Zn^II^ atom is four-coordinated by two N atoms from two 5-*m*-tolyl-1,3,4-thia­diazol-2-ylamine ligands and two Cl anions in a distorted tetra­hedral geometry. Intra­molecular N—H⋯N, N—H⋯Cl and C—H⋯S hydrogen bonds result in the formation of one planar and one non-planar five-membered, one non-planar six-membered and one non-planar seven-membered ring. The six- and seven-membered rings have twist conformations, while the non-planar five-membered ring adopts an envelope conformation with the S atom displaced by 0.541 (3) Å from the plane of the other ring atoms. The planar five-membered ring is oriented at dihedral angles of 1.74 (3) and 1.08 (3)°, respectively, with respect to the adjacent aromatic and thia­diazole rings. In the crystal structure, inter­molecular N—H⋯Cl hydrogen bonds link the mol­ecules into a three-dimensional network.

## Related literature

For general background, see: Alzuet *et al.* (2003 ▶); Shen *et al.* (2004 ▶). For ring puckering parameters, see: Cremer & Pople (1975 ▶).
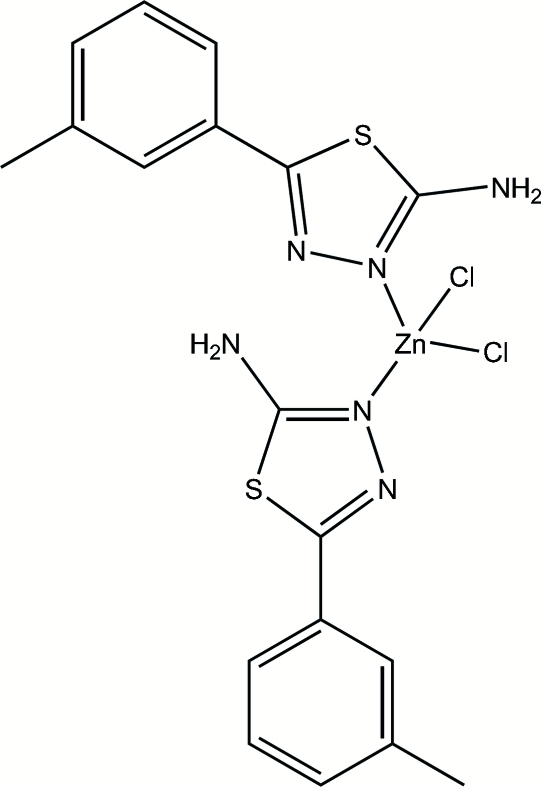


## Experimental

### 

#### Crystal data

[ZnCl_2_(C_9_H_9_N_3_S)_2_]
*M* *_r_* = 518.77
Monoclinic, 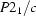

*a* = 10.826 (2) Å
*b* = 11.233 (2) Å
*c* = 17.892 (4) Å
β = 90.10 (3)°
*V* = 2175.8 (7) Å^3^
*Z* = 4
Mo *K*α radiation
μ = 1.58 mm^−1^
*T* = 298 (2) K
0.20 × 0.10 × 0.10 mm

#### Data collection

Enraf–Nonius CAD-4 diffractometer
Absorption correction: ψ scan (North *et al.*, 1968 ▶) *T*_min_ = 0.742, *T*_max_ = 0.858
3917 measured reflections
3917 independent reflections
2310 reflections with *I* > 2σ(*I*)
3 standard reflections frequency: 120 min intensity decay: none

#### Refinement

*R*[*F* ^2^ > 2σ(*F* ^2^)] = 0.086
*wR*(*F* ^2^) = 0.211
*S* = 1.02
3917 reflections
256 parameters
H-atom parameters constrained
Δρ_max_ = 0.62 e Å^−3^
Δρ_min_ = −0.97 e Å^−3^

### 

Data collection: *CAD-4 Software* (Enraf–Nonius, 1989 ▶); cell refinement: *CAD-4 Software*; data reduction: *XCAD4* (Harms & Wocadlo, 1995 ▶); program(s) used to solve structure: *SHELXS97* (Sheldrick, 2008 ▶); program(s) used to refine structure: *SHELXL97* (Sheldrick, 2008 ▶); molecular graphics: *SHELXTL* (Sheldrick, 2008 ▶); software used to prepare material for publication: *SHELXTL*.

## Supplementary Material

Crystal structure: contains datablocks global, I. DOI: 10.1107/S160053680801057X/hk2454sup1.cif

Structure factors: contains datablocks I. DOI: 10.1107/S160053680801057X/hk2454Isup2.hkl

Additional supplementary materials:  crystallographic information; 3D view; checkCIF report

## Figures and Tables

**Table:** 

Zn—Cl1	2.223 (3)
Zn—Cl2	2.270 (3)
Zn—N2	2.056 (6)
Zn—N5	2.089 (8)

**Table:** 

N2—Zn—N5	111.8 (3)
N2—Zn—Cl1	116.5 (2)
N5—Zn—Cl1	105.8 (2)
N2—Zn—Cl2	105.5 (2)
N5—Zn—Cl2	101.7 (2)
Cl1—Zn—Cl2	114.67 (12)

**Table 2:** Hydrogen-bond geometry (Å, °)

*D*—H⋯*A*	*D*—H	H⋯*A*	*D*⋯*A*	*D*—H⋯*A*
N3—H3*B*⋯N4	0.86	2.01	2.864 (10)	174
N3—H3*C*⋯Cl2^i^	0.86	2.53	3.332 (8)	156
N6—H6*B*⋯Cl1	0.86	2.53	3.320 (9)	152
N6—H6*C*⋯Cl2^ii^	0.86	2.60	3.345 (8)	146
C5—H5*A*⋯S1	0.93	2.62	3.040 (10)	108
C10—H10*C*⋯Cl2^iii^	0.96	2.68	3.599 (12)	161
C16—H16*A*⋯S2	0.93	2.85	3.184 (10)	103

Symmetry codes: (i) 
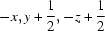
; (ii) 
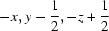
; (iii) 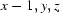.

**Fig. 1.**
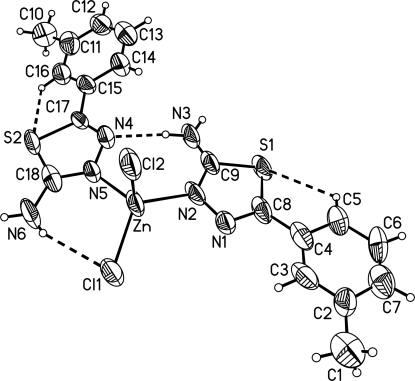
The molecular structure of the title molecule, with the atom-numbering scheme. Displacement ellipsoids are drawn at the 50% probability level. Hydrogen bonds are shown as dashed lines.

**Fig. 2.**
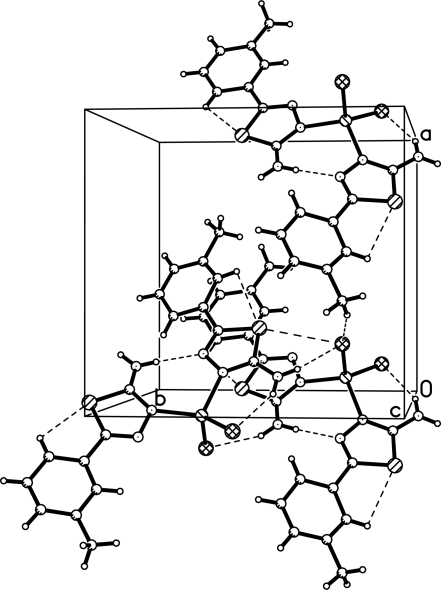
A partial packing diagram of (I). Hydrogen bonds are shown as dashed lines.

## supplementary crystallographic information

### Comment

As a series of superior ligands, thiadiazoles and their derivatives can coordinate to many metal ions with nitrogen or sulfur atoms of the five-membered ring. In particular N,N'-linkage ligands, such as 1,3,4-thiadiazoles, are very versatile compounds that are able to bridge a wide range of inter-metallic separations through two close adjacent N donors (Alzuet *et al.*,  2003). These complexes have received considerable attention in the past few years, due to their certain antibacterial and antifungal activities (Shen *et al.*,  2004).

In the molecule of (I), (Fig. 1), Zn^II^ atom is four-coordinated by two N atoms from two 5-*m*-tolyl-[1,3,4]thiadiazol-2-ylamine ligands and two Cl anions. It has a distorted tetrahedral coordination geometry (Table 1). Rings A (C2-C7), B (N1/N2/S1/C8/C9), C (N4/N5/S2/C17/C18) and D (C11-C16) are, of course, planar. The dihedral angles between them are A/B = 1.87 (3)°, A/C = 14.55 (3)°, A/D = 27.98 (4)°, B/C = 14.63 (4)°, B/D = 28.43 (3)° and C/D = 14.21 (3)°. The intramolecular N-H···N, N-H···Cl and C-H···S hydrogen bonds (Table 2) result in the formation of one planar F (S1/C4/C5/H5A/C8) and one non-planar G (S2/C15-C17/H16A) five-membered, one non-planar H (Zn/Cl1/N5/N6/H6B/C18) six-membered and one non-planar E (Zn/N2-N5/H3B/C9) seven-membered rings. Ring F is oriented with respect to the adjacent rings A and B at dihedral angles of A/F = 1.74 (3)° and B/F = 1.08 (3)°. So, rings A, B and F are nearly coplanar. Rings E and H have twisted conformations, having total puckering amplitudes, Q_T_, of 0.712 (3) and 0.424 (2) Å, respectively (Cremer & Pople, 1975). Ring G adopts envelope conformation with sulfur atom displaced by -0.541 (3) Å from the plane of the other ring atoms.

In the crystal structure, intermolecular N-H···Cl hydrogen bonds (Table 2) link the molecules to form a three-dimensional network (Fig. 2), in which they may be effective in the stabilization of the structure.

### Experimental

For the preparation of the title compound, the solution of ZnCl_2_ (0.5 mmol) in ethanol (20 ml) was added slowly to a solution of 5-*m*-tolyl-[1,3,4]- thiadiazol-2-ylamine (1.0 mmol) in ethanol (20 ml), and then heated under reflux for 2 h. The reaction mixture was left to cool to room temperature, and then filtered. The solid was recrystallized from ethanol to give the title compound, (I), (m.p. 453 K). Crystals of (I) suitable for X-ray analysis were obtained by slow evaporation of an acetone solution.

### Refinement

H atoms were positioned geometrically, with N-H = 0.86 Å (for NH_2_) and C-H = 0.93 and 0.96 Å for aromatic and methyl H, respectively, and constrained to ride on their parent atoms with U_iso_(H) = xU_eq_(C,N), where x = 1.5 for methyl H, and x = 1.2 for all other H atoms.

### Crystal data

**Table:**

[ZnCl_2_(C_9_H_9_N_3_S)_2_]	*F*_000_ = 1056
*M**_r_* = 518.77	*D*_x_ = 1.584 Mg m^−^^3^
Monoclinic, *P*2_1_/*c*	Melting point: 453 K
Hall symbol: -P 2ybc	Mo *K*α radiation λ = 0.71073 Å
*a* = 10.826 (2) Å	Cell parameters from 25 reflections
*b* = 11.233 (2) Å	θ = 10–13º
*c* = 17.892 (4) Å	µ = 1.58 mm^−^^1^
β = 90.10 (3)º	*T* = 298 (2) K
*V* = 2175.8 (7) Å^3^	Block, yellow
*Z* = 4	0.20 × 0.10 × 0.10 mm

### Data collection

**Table:**

Enraf–Nonius CAD-4 diffractometer	*R*_int_ = 0.0000
Radiation source: fine-focus sealed tube	θ_max_ = 25.2º
Monochromator: graphite	θ_min_ = 1.9º
*T* = 298(2) K	*h* = −12→12
ω/2θ scans	*k* = 0→13
Absorption correction: ψ scan(North *et al.*, 1968)	*l* = 0→21
*T*_min_ = 0.742, *T*_max_ = 0.858	3 standard reflections
3917 measured reflections	every 120 min
3917 independent reflections	intensity decay: none
2310 reflections with *I* > 2σ(*I*)	

### Refinement

**Table:**

Refinement on *F*^2^	Secondary atom site location: difference Fourier map
Least-squares matrix: full	Hydrogen site location: inferred from neighbouring sites
*R*[*F*^2^ > 2σ(*F*^2^)] = 0.086	H-atom parameters constrained
*wR*(*F*^2^) = 0.211	*w* = 1/[σ^2^(*F*_o_^2^) + (0.05*P*)^2^ + 19*P*] where *P* = (*F*_o_^2^ + 2*F*_c_^2^)/3
*S* = 1.02	(Δ/σ)_max_ < 0.001
3917 reflections	Δρ_max_ = 0.62 e Å^−^^3^
256 parameters	Δρ_min_ = −0.97 e Å^−^^3^
Primary atom site location: structure-invariant direct methods	Extinction correction: none

### Special details

**Table:**

Geometry. All e.s.d.'s (except the e.s.d. in the dihedral angle between two l.s. planes) are estimated using the full covariance matrix. The cell e.s.d.'s are taken into account individually in the estimation of e.s.d.'s in distances, angles and torsion angles; correlations between e.s.d.'s in cell parameters are only used when they are defined by crystal symmetry. An approximate (isotropic) treatment of cell e.s.d.'s is used for estimating e.s.d.'s involving l.s. planes.
Refinement. Refinement of F^2^ against ALL reflections. The weighted R-factor wR and goodness of fit S are based on F^2^, conventional R-factors R are based on F, with F set to zero for negative F^2^. The threshold expression of F^2^ > 2sigma(F^2^) is used only for calculating R-factors(gt) etc. and is not relevant to the choice of reflections for refinement. R-factors based on F^2^ are statistically about twice as large as those based on F, and R- factors based on ALL data will be even larger.

### Fractional atomic coordinates and isotropic or equivalent isotropic displacement parameters (Å^2^)

**Table:**

	*x*	*y*	*z*	*U*_iso_*/*U*_eq_	
Zn	0.06433 (12)	0.26059 (8)	0.14033 (6)	0.0679 (4)	
Cl1	0.1115 (3)	0.1322 (2)	0.05026 (15)	0.0820 (8)	
Cl2	0.1997 (3)	0.26430 (19)	0.23734 (15)	0.0794 (8)	
S1	0.0144 (3)	0.65934 (18)	0.09796 (14)	0.0734 (8)	
S2	−0.2594 (3)	0.06983 (19)	0.25635 (15)	0.0797 (9)	
N1	0.1364 (8)	0.4722 (6)	0.0595 (4)	0.065 (2)	
N2	0.0424 (7)	0.4353 (6)	0.1087 (4)	0.065 (2)	
N3	−0.1161 (10)	0.5103 (6)	0.1810 (5)	0.083 (3)	
H3B	−0.1321	0.4410	0.1990	0.099*	
H3C	−0.1588	0.5710	0.1948	0.099*	
N4	−0.1619 (7)	0.2723 (6)	0.2312 (4)	0.0616 (19)	
N5	−0.0944 (7)	0.1933 (6)	0.1914 (4)	0.0612 (19)	
N6	−0.0809 (9)	−0.0115 (6)	0.1629 (5)	0.088 (3)	
H6B	−0.0187	0.0009	0.1341	0.106*	
H6C	−0.1088	−0.0826	0.1688	0.106*	
C1	0.4997 (13)	0.5883 (12)	−0.1215 (8)	0.114 (5)	
H1B	0.4935	0.5048	−0.1107	0.171*	
H1C	0.5790	0.6171	−0.1058	0.171*	
H1D	0.4903	0.6007	−0.1743	0.171*	
C2	0.4016 (12)	0.6533 (9)	−0.0812 (6)	0.082 (3)	
C3	0.3158 (11)	0.5948 (9)	−0.0373 (6)	0.080 (3)	
H3A	0.3226	0.5125	−0.0335	0.096*	
C4	0.2196 (12)	0.6500 (8)	0.0019 (5)	0.077 (3)	
C5	0.2031 (9)	0.7744 (8)	−0.0049 (6)	0.068 (3)	
H5A	0.1390	0.8141	0.0191	0.081*	
C6	0.2915 (11)	0.8363 (10)	−0.0513 (7)	0.085 (3)	
H6A	0.2846	0.9181	−0.0579	0.102*	
C7	0.3826 (13)	0.7774 (10)	−0.0845 (7)	0.086 (3)	
H7A	0.4386	0.8218	−0.1123	0.104*	
C8	0.1285 (11)	0.5868 (7)	0.0515 (5)	0.071 (3)	
C9	−0.0319 (12)	0.5226 (7)	0.1353 (6)	0.068 (3)	
C10	−0.6334 (10)	0.2481 (11)	0.4112 (7)	0.092	
H10A	−0.6745	0.2995	0.4461	0.138*	
H10B	−0.6150	0.1735	0.4350	0.138*	
H10C	−0.6861	0.2344	0.3689	0.138*	
C11	−0.5168 (9)	0.3049 (9)	0.3860 (6)	0.069 (2)	
C12	−0.4872 (10)	0.4201 (9)	0.4035 (5)	0.071 (2)	
H12A	−0.5399	0.4636	0.4341	0.085*	
C13	−0.3872 (10)	0.4703 (9)	0.3782 (6)	0.079 (3)	
H13A	−0.3711	0.5494	0.3901	0.095*	
C14	−0.3029 (10)	0.4072 (8)	0.3333 (6)	0.071 (3)	
H14A	−0.2292	0.4407	0.3169	0.085*	
C15	−0.3377 (9)	0.2900 (7)	0.3149 (5)	0.060 (2)	
C16	−0.4505 (10)	0.2402 (8)	0.3390 (5)	0.070 (2)	
H16A	−0.4769	0.1658	0.3227	0.084*	
C17	−0.2499 (9)	0.2250 (7)	0.2652 (5)	0.057 (2)	
C18	−0.1351 (9)	0.0809 (8)	0.1990 (6)	0.063 (2)	

### Atomic displacement parameters (Å^2^)

**Table:**

	*U*^11^	*U*^22^	*U*^33^	*U*^12^	*U*^13^	*U*^23^
Zn	0.1219 (10)	0.0225 (5)	0.0593 (7)	0.0009 (5)	−0.0147 (6)	0.0037 (5)
Cl1	0.134 (2)	0.0377 (12)	0.0743 (17)	0.0062 (14)	−0.0046 (16)	−0.0073 (11)
Cl2	0.134 (2)	0.0332 (11)	0.0712 (16)	−0.0054 (13)	−0.0253 (15)	0.0062 (11)
S1	0.134 (2)	0.0210 (10)	0.0646 (15)	0.0045 (12)	−0.0170 (15)	0.0042 (10)
S2	0.146 (3)	0.0255 (11)	0.0671 (16)	−0.0043 (13)	−0.0155 (16)	0.0022 (11)
N1	0.114 (7)	0.035 (4)	0.046 (4)	−0.009 (4)	−0.003 (4)	0.001 (3)
N2	0.114 (7)	0.022 (3)	0.059 (5)	0.010 (4)	−0.013 (5)	−0.003 (3)
N3	0.159 (9)	0.024 (4)	0.066 (6)	−0.001 (5)	−0.011 (6)	0.004 (4)
N4	0.084 (5)	0.037 (3)	0.064 (4)	−0.002 (3)	−0.003 (4)	−0.001 (3)
N5	0.095 (5)	0.033 (3)	0.055 (4)	0.004 (3)	−0.002 (4)	0.000 (3)
N6	0.130 (8)	0.025 (4)	0.110 (7)	0.000 (4)	−0.002 (6)	−0.008 (4)
C1	0.130 (11)	0.082 (9)	0.130 (12)	0.000 (8)	0.006 (9)	−0.015 (9)
C2	0.125 (10)	0.058 (6)	0.064 (6)	−0.028 (6)	0.014 (6)	0.005 (5)
C3	0.127 (9)	0.041 (5)	0.072 (7)	0.003 (6)	−0.010 (7)	−0.017 (5)
C4	0.146 (10)	0.037 (5)	0.047 (5)	−0.014 (6)	−0.032 (6)	0.004 (4)
C5	0.074 (6)	0.040 (5)	0.090 (7)	0.000 (4)	−0.013 (5)	0.007 (5)
C6	0.094 (8)	0.049 (6)	0.112 (9)	−0.024 (6)	−0.020 (7)	0.029 (6)
C7	0.119 (10)	0.055 (6)	0.085 (8)	−0.006 (7)	−0.020 (7)	0.019 (6)
C8	0.131 (9)	0.030 (4)	0.052 (5)	−0.009 (5)	−0.030 (6)	0.008 (4)
C9	0.129 (9)	0.024 (4)	0.051 (6)	−0.002 (5)	−0.022 (6)	0.002 (4)
C10	0.092	0.092	0.092	0.000	0.000	0.000
C11	0.072 (5)	0.059 (5)	0.077 (6)	−0.006 (4)	−0.020 (4)	0.004 (4)
C12	0.084 (6)	0.060 (5)	0.069 (6)	0.007 (4)	−0.007 (4)	−0.007 (4)
C13	0.091 (6)	0.056 (5)	0.090 (6)	0.001 (4)	−0.004 (5)	−0.013 (5)
C14	0.094 (6)	0.043 (4)	0.076 (6)	−0.002 (4)	−0.002 (5)	−0.002 (4)
C15	0.094 (5)	0.037 (4)	0.049 (5)	0.017 (4)	−0.011 (4)	−0.004 (4)
C16	0.105 (6)	0.045 (4)	0.061 (5)	−0.003 (4)	−0.014 (4)	0.002 (4)
C17	0.094 (5)	0.034 (4)	0.043 (4)	−0.003 (4)	−0.006 (4)	−0.007 (3)
C18	0.074 (5)	0.037 (4)	0.077 (5)	0.003 (4)	−0.025 (4)	−0.004 (4)

### Geometric parameters (Å, °)

**Table:**

Zn—Cl1	2.223 (3)	C3—H3A	0.9300
Zn—Cl2	2.270 (3)	C4—C5	1.414 (12)
Zn—N2	2.056 (6)	C4—C8	1.505 (14)
Zn—N5	2.089 (8)	C5—C6	1.446 (14)
S1—C8	1.698 (11)	C5—H5A	0.9300
S1—C9	1.748 (9)	C6—C7	1.328 (15)
S2—C18	1.699 (11)	C6—H6A	0.9300
S2—C17	1.753 (8)	C7—H7A	0.9300
N1—N2	1.410 (8)	C9—N3	1.234 (13)
N1—C8	1.298 (11)	C10—C11	1.485 (14)
N2—C9	1.355 (12)	C10—H10A	0.9600
N3—H3B	0.8600	C10—H10B	0.9600
N3—H3C	0.8600	C10—H10C	0.9600
N4—C17	1.250 (11)	C11—C16	1.324 (11)
N5—N4	1.353 (10)	C11—C12	1.370 (13)
N5—C18	1.344 (11)	C12—C13	1.302 (14)
N6—H6B	0.8600	C12—H12A	0.9300
N6—H6C	0.8600	C13—C14	1.409 (13)
C1—C2	1.478 (16)	C13—H13A	0.9300
C1—H1B	0.9600	C14—C15	1.408 (12)
C1—H1C	0.9600	C14—H14A	0.9300
C1—H1D	0.9600	C15—C16	1.411 (13)
C2—C3	1.383 (14)	C15—C17	1.494 (12)
C2—C7	1.411 (15)	C16—H16A	0.9300
C3—C4	1.402 (15)	C18—N6	1.357 (12)
			
N2—Zn—N5	111.8 (3)	C7—C6—H6A	119.7
N2—Zn—Cl1	116.5 (2)	C5—C6—H6A	119.7
N5—Zn—Cl1	105.8 (2)	C6—C7—C2	125.6 (12)
N2—Zn—Cl2	105.5 (2)	C6—C7—H7A	117.2
N5—Zn—Cl2	101.7 (2)	C2—C7—H7A	117.2
Cl1—Zn—Cl2	114.67 (12)	N1—C8—C4	119.3 (10)
C8—S1—C9	88.6 (5)	N1—C8—S1	118.0 (8)
C18—S2—C17	86.3 (4)	C4—C8—S1	122.7 (7)
C8—N1—N2	108.2 (8)	N3—C9—N2	126.2 (9)
C9—N2—N1	115.8 (7)	N3—C9—S1	124.4 (8)
C9—N2—Zn	131.5 (6)	N2—C9—S1	109.4 (8)
N1—N2—Zn	111.7 (5)	C11—C10—H10A	109.5
C9—N3—H3B	120.0	C11—C10—H10B	109.5
C9—N3—H3C	120.0	H10A—C10—H10B	109.5
H3B—N3—H3C	120.0	C11—C10—H10C	109.5
C17—N4—N5	112.9 (7)	H10A—C10—H10C	109.5
C18—N5—N4	112.7 (8)	H10B—C10—H10C	109.5
C18—N5—Zn	130.8 (7)	C16—C11—C12	122.4 (10)
N4—N5—Zn	116.0 (5)	C16—C11—C10	114.8 (10)
C18—N6—H6B	120.0	C12—C11—C10	122.4 (10)
C18—N6—H6C	120.0	C13—C12—C11	121.7 (10)
H6B—N6—H6C	120.0	C13—C12—H12A	119.2
C2—C1—H1B	109.5	C11—C12—H12A	119.2
C2—C1—H1C	109.5	C12—C13—C14	121.3 (10)
H1B—C1—H1C	109.5	C12—C13—H13A	119.3
C2—C1—H1D	109.5	C14—C13—H13A	119.3
H1B—C1—H1D	109.5	C15—C14—C13	115.5 (10)
H1C—C1—H1D	109.5	C15—C14—H14A	122.3
C3—C2—C7	113.3 (11)	C13—C14—H14A	122.3
C3—C2—C1	121.8 (10)	C14—C15—C16	122.0 (9)
C7—C2—C1	124.9 (11)	C14—C15—C17	115.2 (9)
C2—C3—C4	125.1 (10)	C16—C15—C17	122.7 (8)
C2—C3—H3A	117.5	C11—C16—C15	116.6 (9)
C4—C3—H3A	117.5	C11—C16—H16A	121.7
C3—C4—C5	119.2 (10)	C15—C16—H16A	121.7
C3—C4—C8	125.1 (9)	N4—C17—C15	124.6 (8)
C5—C4—C8	115.7 (10)	N4—C17—S2	115.1 (7)
C4—C5—C6	116.1 (10)	C15—C17—S2	120.2 (7)
C4—C5—H5A	121.9	N5—C18—N6	121.9 (9)
C6—C5—H5A	121.9	N5—C18—S2	113.0 (7)
C7—C6—C5	120.7 (10)	N6—C18—S2	125.1 (7)
			
N5—Zn—N2—C9	25.7 (10)	C9—S1—C8—C4	−179.4 (8)
Cl1—Zn—N2—C9	147.6 (8)	N1—N2—C9—N3	−177.0 (10)
Cl2—Zn—N2—C9	−84.0 (9)	Zn—N2—C9—N3	−9.3 (16)
N5—Zn—N2—N1	−166.2 (5)	N1—N2—C9—S1	0.3 (10)
Cl1—Zn—N2—N1	−44.4 (6)	Zn—N2—C9—S1	167.9 (5)
Cl2—Zn—N2—N1	84.1 (5)	C8—S1—C9—N3	176.8 (10)
N2—Zn—N5—C18	156.5 (8)	C8—S1—C9—N2	−0.5 (7)
Cl1—Zn—N5—C18	28.7 (9)	C16—C11—C12—C13	−4.5 (13)
Cl2—Zn—N5—C18	−91.4 (8)	C10—C11—C12—C13	−177.0 (10)
N2—Zn—N5—N4	−32.2 (7)	C11—C12—C13—C14	−1.7 (13)
Cl1—Zn—N5—N4	−160.0 (6)	C12—C13—C14—C15	3.2 (14)
Cl2—Zn—N5—N4	79.9 (6)	C13—C14—C15—C16	0.9 (14)
C8—N1—N2—C9	0.2 (11)	C13—C14—C15—C17	178.1 (8)
C8—N1—N2—Zn	−169.9 (6)	C12—C11—C16—C15	8.2 (14)
C18—N5—N4—C17	−1.7 (12)	C10—C11—C16—C15	−178.8 (9)
Zn—N5—N4—C17	−174.6 (6)	C14—C15—C16—C11	−6.5 (14)
C7—C2—C3—C4	−1.0 (17)	C17—C15—C16—C11	176.6 (8)
C1—C2—C3—C4	−179.3 (11)	N5—N4—C17—C15	178.4 (8)
C2—C3—C4—C5	2.6 (17)	N5—N4—C17—S2	2.6 (11)
C2—C3—C4—C8	−178.1 (10)	C14—C15—C17—N4	−11.5 (14)
C3—C4—C5—C6	−1.8 (15)	C16—C15—C17—N4	165.6 (9)
C8—C4—C5—C6	178.9 (9)	C14—C15—C17—S2	164.0 (7)
C4—C5—C6—C7	−0.4 (16)	C16—C15—C17—S2	−18.8 (12)
C5—C6—C7—C2	2(2)	C18—S2—C17—N4	−2.2 (8)
C3—C2—C7—C6	−1.5 (19)	C18—S2—C17—C15	−178.2 (8)
C1—C2—C7—C6	176.8 (13)	N4—N5—C18—N6	179.1 (9)
C3—C4—C8—N1	−0.5 (15)	Zn—N5—C18—N6	−9.4 (14)
C5—C4—C8—N1	178.8 (9)	N4—N5—C18—S2	0.0 (10)
C3—C4—C8—S1	179.6 (8)	Zn—N5—C18—S2	171.5 (5)
C5—C4—C8—S1	−1.1 (13)	C17—S2—C18—N5	1.1 (7)
C9—S1—C8—N1	0.7 (8)	C17—S2—C18—N6	−178.0 (9)

### Hydrogen-bond geometry (Å, °)

**Table:**

*D*—H···*A*	*D*—H	H···*A*	*D*···*A*	*D*—H···*A*
N3—H3B···N4	0.86	2.01	2.864 (10)	174
N3—H3C···Cl2^i^	0.86	2.53	3.332 (8)	156
N6—H6B···Cl1	0.86	2.53	3.320 (9)	152
N6—H6C···Cl2^ii^	0.86	2.60	3.345 (8)	146
C5—H5A···S1	0.93	2.62	3.040 (10)	108
C10—H10C···Cl2^iii^	0.96	2.68	3.599 (12)	161
C16—H16A···S2	0.93	2.85	3.184 (10)	103

Symmetry codes: (i) −*x*, *y*+1/2, −*z*+1/2; (ii) −*x*, *y*−1/2, −*z*+1/2; (iii) *x*−1, *y*, *z*.

## References

[bb1] Alzuet, G., Borras, J., Estevan, F., Liu-Gonzalez, M. & Sanz-Ruiz, F. (2003). *Inorg. Chim. Acta*, **343**, 56–60.

[bb2] Cremer, D. & Pople, J. A. (1975). *J. Am. Chem. Soc.* **97**, 1354–1358.

[bb3] Enraf–Nonius (1989). *CAD-4 Software* Enraf–Nonius, Delft, The Netherlands.

[bb4] Harms, K. & Wocadlo, S. (1995). *XCAD4* University of Marburg, Germany.

[bb5] North, A. C. T., Phillips, D. C. & Mathews, F. S. (1968). *Acta Cryst.* A**24**, 351–359.

[bb6] Sheldrick, G. M. (2008). *Acta Cryst.* A**64**, 112–122. 10.1107/S0108767307043930 18156677

[bb7] Shen, X.-Q., Zhong, H.-J. & Zheng, H. (2004). *Polyhedron*, **23**, 1851–1857.

